# The Thiol Reductase Activity of YUCCA6 Mediates Delayed Leaf Senescence by Regulating Genes Involved in Auxin Redistribution

**DOI:** 10.3389/fpls.2016.00626

**Published:** 2016-05-09

**Authors:** Joon-Yung Cha, Mi R. Kim, In J. Jung, Sun B. Kang, Hee J. Park, Min G. Kim, Dae-Jin Yun, Woe-Yeon Kim

**Affiliations:** ^1^Division of Applied Life Science (BK21 Plus), Plant Molecular Biology and Biotechnology Research Center, Institute of Agriculture and Life Sciences, Gyeongsang National UniversityJinju, South Korea; ^2^College of Pharmacy, Research Institute of Pharmaceutical Science, Plant Molecular Biology and Biotechnology Research Center, Gyeongsang National UniversityJinju, South Korea

**Keywords:** auxin, reactive oxygen species, redox signaling, senescence, thiol reductase

## Abstract

Auxin, a phytohormone that affects almost every aspect of plant growth and development, is biosynthesized from tryptophan via the tryptamine, indole-3-acetamide, indole-3-pyruvic acid, and indole-3-acetaldoxime pathways. YUCCAs (YUCs), flavin monooxygenase enzymes, catalyze the conversion of indole-3-pyruvic acid (IPA) to the auxin (indole acetic acid). *Arabidopsis thaliana* YUC6 also exhibits thiol-reductase and chaperone activity *in vitro*; these activities require the highly conserved Cys-85 and are essential for scavenging of toxic reactive oxygen species (ROS) in the drought tolerance response. Here, we examined whether the YUC6 thiol reductase activity also participates in the delay in senescence observed in *YUC6*-overexpressing (YUC6*-*OX) plants. *YUC6* overexpression delays leaf senescence in natural and dark-induced senescence conditions by reducing the expression of *SENESCENCE-ASSOCIATED GENE 12* (*SAG12*). ROS accumulation normally occurs during senescence, but was not observed in the leaves of YUC6-OX plants; however, ROS accumulation was observed in YUC6-OX^C85S^ plants, which overexpress a mutant YUC6 that lacks thiol reductase activity. We also found that YUC6-OX plants, but not YUC6-OX^C85S^ plants, show upregulation of three genes encoding NADPH-dependent thioredoxin reductases (*NTRA, NTRB*, and *NTRC*), and *GAMMA-GLUTAMYLCYSTEINE SYNTHETASE 1* (*GSH1*), encoding an enzyme involved in redox signaling. We further determined that excess ROS accumulation caused by methyl viologen treatment or decreased glutathione levels caused by buthionine sulfoximine treatment can decrease the levels of auxin eﬄux proteins such as PIN2-4. The expression of *PIN*s is also reduced in YUC6-OX plants. These findings suggest that the thiol reductase activity of YUC6 may play an essential role in delaying senescence via the activation of genes involved in redox signaling and auxin availability.

## Introduction

Plants undergo senescence to mobilize nutrients and remove unneeded organs. Senescing leaves break down chlorophyll and degrade macromolecules for nutrient translocation; the reactive oxygen species (ROS)-detoxifying system also breaks down. Various phytohormones and environmental conditions interact to regulate senescence (Lim et al., 2007). Exogenous application of ethylene, abscisic acid (ABA), or jasmonic acid (JA) triggers leaf senescence (Grbić and Bleecker, 1995; Weaver et al., 1998; He et al., 2002). Endogenous salicylic acid (SA) also increases in senescing leaves (Khan et al., 2014). By contrast, treatment with cytokinins or auxin delays senescence (Noodén et al., 1990; Kim et al., 2011). In addition, detoxification systems such as antioxidant enzymes delay senescence (Ye et al., 2000; Lim et al., 2007). The developmental shift to senescence in plants is determined by the induction of Senescence-Associated Genes (*SAGs*), which are differentially expressed in response to treatment with phytohormones associated with senescence (Weaver et al., 1998; Gepstein et al., 2003).

Auxin regulates diverse aspects of plant growth and development, including apical dominance, tropisms, root, and shoot development, vascular differentiation, and embryo patterning (Vanneste and Friml, 2009). Auxin is synthesized via four distinct tryptophan (Trp)-dependent pathways: the tryptamine (TAM), indole-3-acetamide (IAM), indole-3-pyruvic acid (IPA), and indole-3-acetaldoxime (IAOx) pathways (Quittenden et al., 2009; Zhao, 2012). YUCCA (YUC) proteins, which are members of the plant flavin monooxygenase (FMO) family, catalyze the conversion of IPA to indole acetic acid (IAA) via the action of Trp aminotransferase (TAA1/TAR1/TAR2; Stepanova et al., 2011; Dai et al., 2013). We recently demonstrated that, in addition to its FMO function, YUC6 also acts as a thiol-reductase (TR) involved in auxin biosynthesis and ROS homeostasis (Cha et al., 2015). Independent of the auxin biosynthesis activity of YUC6, its TR activity reduces ROS induction under oxidative and drought stress, thereby increasing stress tolerance. In addition, a cysteine residue (Cys85, based on the YUC6 sequence) that is highly conserved in 11 *Arabidopsis* YUC proteins is essential for TR activity and ROS regulation both *in vitro* and *in vivo*. TR proteins, including NADPH-dependent thioredoxin reductases (NTRs), function as redox proteins involved in the disulfide reduction via dithiol- or monothiol-related mechanisms (Arnér and Holmgren, 2000; Jacquot et al., 2009). Bashandy et al. (2010) demonstrated that mutants affecting NTR (*ntra ntrb*) or glutathione biosynthesis (*cad2*) exhibit disturbed auxin signaling, and *ntra ntrb cad2* triple mutant plants exhibit developmental defects caused by reduced auxin levels and transport. The *Arabidopsis* NADPH-dependent thioredoxin (NTS) and NADPH-dependent glutathione systems (NGS) interact. Recent studies suggest that developmental defects caused by the inhibition of NTS and NGS are due to reduced auxin levels and reduced auxin transport.

Overexpression of *YUC*6 in plants delays leaf senescence by increasing auxin levels (Kim et al., 2011). In the current study, we investigated whether delayed leaf senescence caused by *YUC6* overexpression also requires the TR activity of YUC6. Interestingly, reducing TR activity by mutating Cys85 in YUC6 abolished the delayed leaf senescence of *YUC6*-overexpressing plants by increasing ROS accumulation in senescing leaves. We also found that elevated ROS levels trigger the reduced expression of auxin transporter genes, such as *PINs*, in a TR activity-dependent manner. Therefore, our results suggest that YUC6 mediates delayed leaf senescence by regulating ROS homeostasis and auxin transporters via its TR activity.

## Results

### The Delay of Natural and Dark-Induced Senescence Requires the Thiol Reductase Activity of YUC6

We previously showed that plants overexpressing *YUC6*, carrying the dominant mutant *yuc6-1D*, or transgenic for a *35S:YUC6* construct all exhibit delayed leaf senescence, along with extreme longevity (Kim et al., 2011). The delayed senescence in response to *YUC6* overexpression is accompanied by elevated auxin levels. Recently, we also determined that YUC6 plays a dual role in regulating plant development and reducing stress responses via its FMO and TR activities, respectively (Cha et al., 2015). Therefore, in the present study, we used YUC6-OX^C85S^ plants to investigate whether the TR activity of YUC6 is also necessary for delaying senescence. The YUC6-OX^C85S^ plants overexpress *YUC6* and have a mutation in a conserved cysteine residue (Cys-85); this mutation suppresses the TR activity of YUC6, but does not affect its FMO activity *in vitro*. In addition, the overexpressed YUC6 protein levels and auxin amounts were not significantly different between YUC6-OX and YUC6-OX^C85S^ plants (Cha et al., 2015).

**FIGURE 1:**
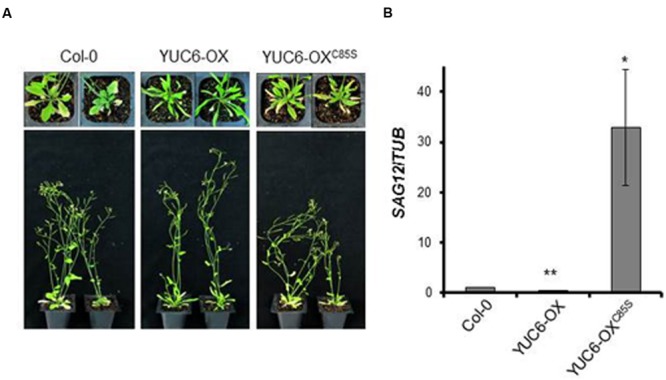
**Overexpression of *YUC6* delays leaf senescence in a TR-activity-dependent manner. (A)** Natural senescence assay. Wild-type (Col-0), YUC6-OX, and YUC6-OX^C85S^ plants were grown in soil and photographed at 40 days after sowing. **(B)** Levels of *SAG12* transcripts. Leaves were harvested at 40 days after sowing. Shown are the mRNA levels of *SAG12*, which were normalized to *TUBULIN* (*TUB*) mRNA levels, as measured by quantitative RT-PCR. Data represent the means ± SE, *n* = 3. ^∗^*P* < 0.05, ^∗∗^*P* < 0.01 compared with *SAG12* expression levels in Col-0; two-tailed Student’s *t*-test; *n* refers to the number of biological replicates.

We first examined the YUC6-OX and YUC6-OX^C85S^ plants under natural senescence conditions. At 40 days after sowing, YUC6-OX plants had fewer senescent leaves than wild-type (Col-0) plants, which is consistent with a previous report (Kim et al., 2011; **Figure 1A**). However, YUC6-OX^C85S^ and wild-type plants had more senescent leaves than YUC6-OX plants. To confirm that the delayed leaf senescence phenotype was caused by *YUC6* overexpression at the molecular level, we examined the expression of a representative downstream gene, *SENESCENCE-ASSOCIATED GENE 12* (*SAG12*), which is upregulated during senescence. As shown in **Figure 1B**, *SAG12* transcript levels were significantly lower in YUC6-OX than in wild-type and YUC6-OX^C85S^ plants (**Figure 1**).

**FIGURE 2:**
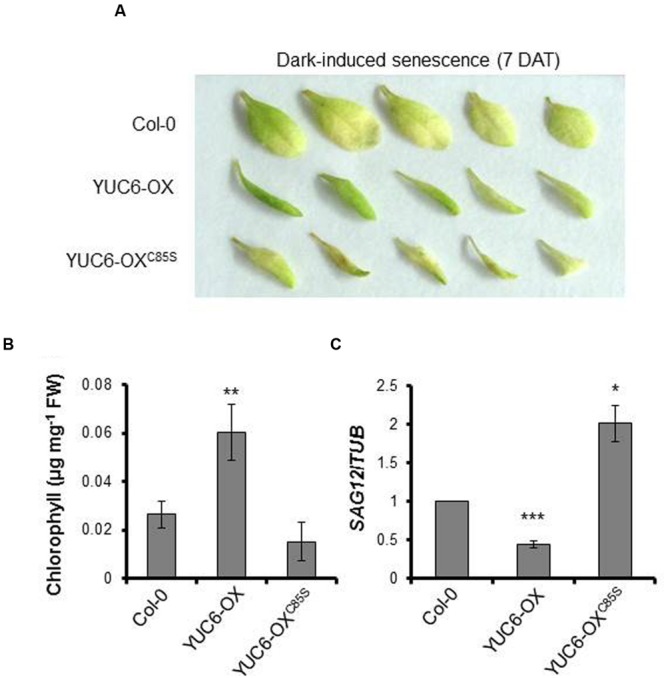
**Dark-induced senescence.** Wild-type (Col-0), YUC6-OX, and YUC6-OX^C85S^ plants (3.5 weeks old) were exposed to constant dark conditions for 7 days. The third or fourth leaves were detached and photographed at 7 days after dark treatment (7 DAT), as shown in **(A)**. **(B)** Chlorophyll contents. Leaves from plants treated to induce dark-induced senescence were harvested as described in **(A)** and their chlorophyll contents were measured. Data represent the means ± SE, *n* = 3. ^∗∗^*P* < 0.01 compared with the chlorophyll contents in Col-0; two-tailed Student’s *t*-test; *n* refers to the number of biological replicates. **(C)** Expression of *SAG12*. Shown are the mRNA levels of *SAG12*, which were relatively normalized to *TUBULIN* (*TUB*) mRNA levels, as measured by quantitative RT-PCR. Data represent the means ± SE, *n* = 3. ^∗^*P* < 0.05, ^∗∗∗^*P* < 0.001 compared with *SAG12* expression levels in Col-0; two-tailed Student’s *t*-test; *n* refers to number of biological replicates.

To further investigate the importance of the TR activity of YUC6 during leaf senescence, 2-week-old plants were maintained under dark conditions to artificially induce senescence (Kim et al., 2011). At 7 days after dark treatment (7 DAT), leaves of YUC6-OX plants were greener than those of the wild type and YUC6-OX^C85S^, whereas YUC6-OX^C85S^ leaves were either light green or brownish in color, indicating that they were undergoing senescence (**Figure 2A**). We measured the chlorophyll contents in the leaves to monitor the progression of senescence. As shown in **Figure 2B**, chlorophyll contents were significantly higher in YUC6-OX plants compared to wild-type and YUC6-OX^C85S^ plants, consistent with the phenotypes shown in **Figure 2A**. The leaf senescence phenotypes of dark-treated plant samples were confirmed at the molecular level by measuring the expression of *SAG12*. Consistent with the results under natural senescence conditions, the expression of *SAG12* in YUC6-OX^C85S^ plants was significantly higher than in wild-type and YUC6-OX plants under dark-induced senescence (**Figure 2C**). We previously determined that the lack of the TR activity of YUC6 in YUC6-OX^C85S^ (due to the mutation of Cys85) does not abolish IAA production *in planta* (Cha et al., 2015). Thus, these data suggest that the TR activity of YUC6 may be also essential for delaying leaf senescence.

### The TR Activity of YUC6 Represses H_2_O_2_ Accumulation in Senescence

**FIGURE 3:**
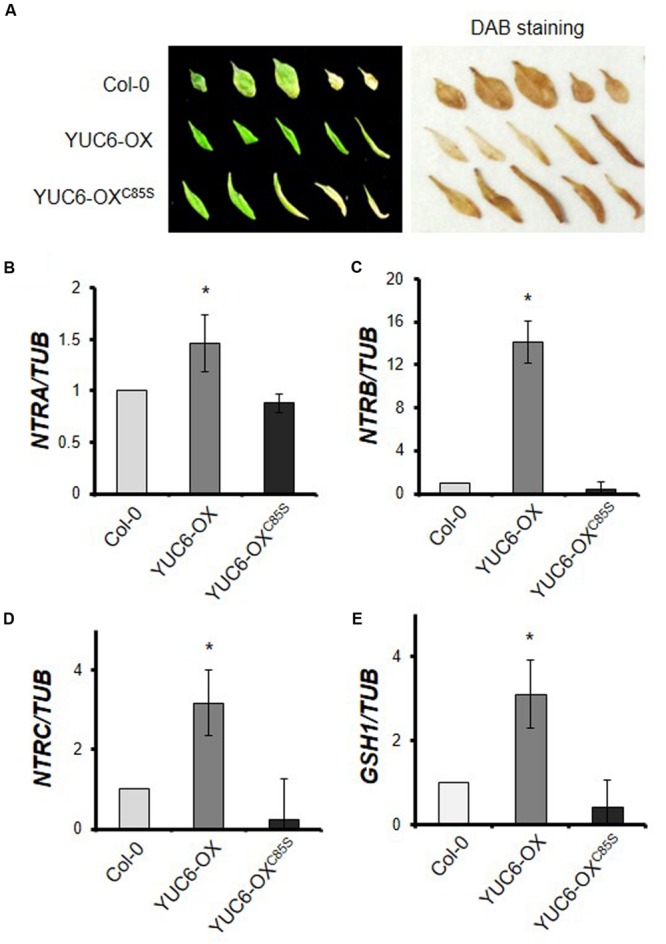
**H_2_O_2_ accumulation and expression patterns of genes involved in the redox system (NTS/NGS) during senescence. (A)** H_2_O_2_ accumulation detected by DAB staining. Wild-type (Col-0), YUC6-OX, and YUC6-OX^C85S^ plants were grown in soil under natural senescence conditions as shown in **Figure 1**. Leaves from 40-day-old plants were detached and photographed as shown on the left. The leaves were stained with DAB solution for 4 h and H_2_O_2_ accumulation was visualized as dark brown coloring in leaves on the right. **(B–E)** Levels of transcripts of genes involved in redox systems. Third or fourth rosette leaves of 3.5-week-old plants were harvested and the expression of genes involved in the NADPH-dependent thioredoxin system (NTS) and glutathione system (NGS) was quantitatively analyzed using RT-PCR. The mRNA levels of *NTRA* **(B)**, *NTRB* **(C)**, *NTRC* **(D)**, and *GSH1* **(E)** were normalized to *TUB* mRNA levels. Data represent the means ± SE, *n* = 3. ^∗^*P* < 0.05 compared with each gene expression levels in Col-0; two-tailed Student’s *t*-test; *n* refers to number of biological replicates.

Plant hormones regulate senescence in a complex manner. The production of ROS, such as free radicals and hydrogen peroxide (H_2_O_2_), also triggers senescence (Strother, 1988; Lim et al., 2007). We recently showed that YUC6 is involved in controlling ROS homeostasis to protect plants from oxidative and drought stress via its TR activity (Cha et al., 2015). To determine whether the regulation of ROS levels by the TR activity of YUC6 affects leaf senescence, we examined H_2_O_2_ accumulation in senescent leaves. Naturally senescent leaves (shown in **Figure 1A**) were detached and ROS accumulation was visualized by staining with 3,3′-diaminobenzidine (DAB; **Figure 3A**). Healthy green leaves of YUC6-OX plants exhibited almost no accumulation of H_2_O_2_; by contrast, high levels of H_2_O_2_ accumulated in both healthy and senescent leaves of wild-type and YUC6-OX^C85S^ plants. ROS homeostasis is maintained during the vegetative stage, but at the beginning of the reproductive stage, this homeostasis shifts and ROS levels increase, initiating senescence (Jing et al., 2008). This increase occurs in conjunction with a reduction in antioxidant enzyme activity during senescence (Ye et al., 2000). Thus, the disruption of redox homeostasis can lead to leaf senescence. We previously determined that peroxidase activity is enhanced in *yuc6-1D* and *YUC6*-overexpressing plants in a TR activity-dependent manner, but with no changes in catalase activity (Cha et al., 2015).

In addition, key redox systems such as NTS and NGS are associated with auxin signaling, including regulation of auxin levels and transport (Bashandy et al., 2010). Thus, we examined the transcript levels of three NTR genes, *NTRA, NTRB*, and *NTRC*, as well as *GSH1* (**Figures 3B–E**). Interestingly, whereas the transcript levels of all of these genes were elevated in YUC6-OX plants compared to wild type, changes in transcript levels were not observed in YUC6-OX^C85S^ plants; rather, the transcript levels in these plants were similar to those of wild type. These results suggest that *YUC6* overexpression enhances expression of genes involved in NTS and NGS in a TR activity-dependent manner in YUC6-OX plants and that it also may delay leaf senescence by inhibiting ROS induction.

### ROS Accumulation Reduces Auxin Eﬄux Transporter Levels

**FIGURE 4:**
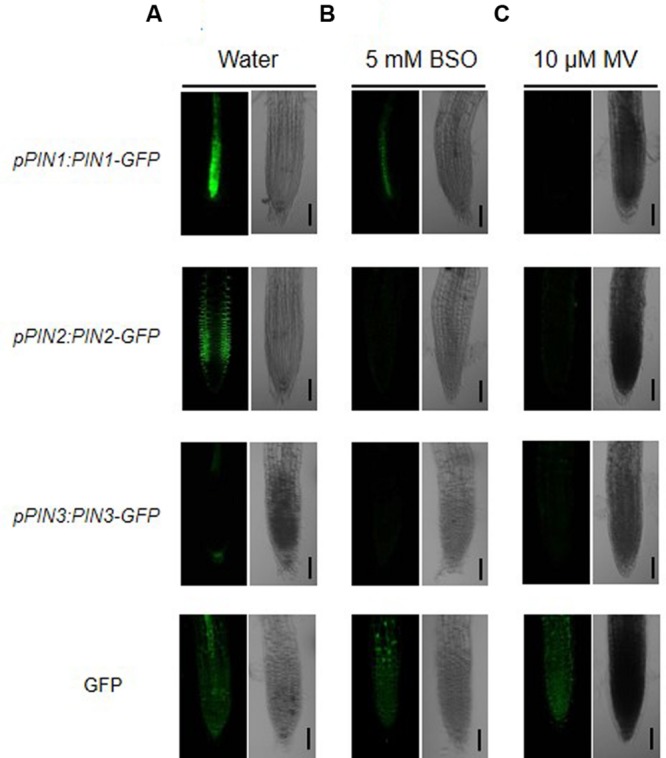
**ROS accumulation reduces expression of auxin transporter genes.** Five-day-old seedlings expressing *pPIN1:PIN1-GFP, pPIN2:PIN2-GFP, pPIN3:PIN3-GFP*, and *35S:GFP* were incubated either in water **(A)**, BSO **(B**, 5 mM), or methyl viologen (MV; **C**, 10 μM) for 12 h as described (Bashandy et al., 2010) with minor modifications. The seedlings were rinsed twice with distilled water and GFP signals were analyzed by confocal microscopy. Bar = 50 μm.

In the absence of NTS and NGS, mutant plants display phenotypes similar to those of auxin-defective mutants. In addition, treatment with buthionine sulfoximine (BSO), an inhibitor of glutathione biosynthesis, triggers decreases in the levels of auxin transporters, such as PIN proteins, at the transcriptional and translational levels (Bashandy et al., 2010). Thus, we examined whether the accumulation of ROS induced by methyl viologen (MV), an oxidative stress agent, also causes a decrease in the levels of PIN proteins. Five-day-old transgenic plants expressing *PIN1-GFP, PIN2-GFP*, and *PIN3-GFP* (which encode PINs with different polar localizations) driven by their native promoters were subjected to BSO and MV treatment (**Figure 4**). As shown in **Figure 4A**, the water-treated control roots showed distinct localization patterns of each PIN-GFP fusion protein, consistent with previous reports (Bashandy et al., 2010; Friml, 2010). However, when the seedlings were subjected to treatment with 5 mM BSO for 12 h, the signals from each PIN-GFP fusion protein were dramatically reduced or absent (**Figure 4B**). These results support the finding of Bashandy et al. (2010) that glutathione availability is important for the expression of PINs. Moreover, we used MV treatment to investigate whether ROS accumulation triggered by oxidative damage also mediates the reduced expression of PIN-GFPs (**Figure 4C**). Consistent with the results of the BSO treatment, all PIN-GFP signals were clearly diminished by MV treatment. These results suggest that excessive ROS accumulation in plant cells reduces the availability of auxin by reducing the levels of auxin eﬄux transporters.

### YUC6 Regulates the Expression of PINs in a TR Activity-Dependent Manner

**FIGURE 5:**
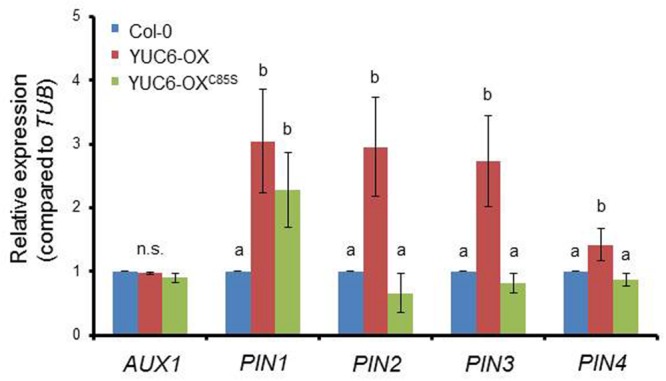
**The reduction in expression of auxin transporter genes requires the TR activity of YUC6.** Third or fourth rosette leaves of 3.5-week-old wild-type (Col-0), YUC6-OX, and YUC6-OX^C85S^ plants were harvested and analyzed using RT-PCR. The mRNA levels of genes encoding auxin influx carrier (AUX1) and eﬄux carriers (PIN1, PIN2, PIN3, and PIN4) were monitored and normalized to *TUB* mRNA levels. Data represent the means ± SE, *n* = 3. Different letters above bars indicate statistically significant differences as determined by one-way ANOVA and Duncan’s HSD, *P* < 0.05. n.s., no significant differences among plants.

We previously reported that the TR activity of YUC6 functions in activating redox systems to scavenge ROS produced under oxidative and drought stresses (Cha et al., 2015). Auxin transport interacts with the glutathione and thioredoxin systems by influencing transcriptional and translational regulation of PIN proteins, thereby regulating physiological responses in plants (Bashandy et al., 2010; Koprivova et al., 2010). Thus, we examined whether the TR activity of YUC6 also influences the expression of auxin transporters by abolishing ROS regulation via the loss of TR activity. Auxin is imported into cells via the AUX1 carrier and is exported by PIN eﬄux proteins, which exhibit polar localization in cells (Friml, 2010). The levels of most *PIN* gene transcripts were higher in YUC6-OX plants compared to wild type, while those in YUC6-OX^C85S^ plants were significantly lower than in YUC6-OX plants, but similar to levels in wild type (**Figure 5**). However, *AUX1* and *PIN1* transcript levels did not differ among lines. Although YUC6-OX and YUC6-OX^C85S^ plants show no difference in auxin levels (Cha et al., 2015), YUC6 affects the expression of auxin eﬄux genes for redistribution, such as *PIN*s, in a TR activity-dependent manner. As ROS levels are important determinants of delayed leaf senescence in *YUC6* overexpressing plants, ROS may influence the mobility and availability of biosynthesized auxin.

## Discussion

Plants regulate their growth and development in response to numerous external stimuli and internal cues, such as various phytohormones. These external and internal factors interact in a complex manner to activate or repress genetic programs in cells throughout the plant’s life (Peleg and Blumwald, 2011). These factors trigger diverse downstream responses depending on the plant’s developmental stage, especially during senescence (Lim et al., 2007). Phytohormones play diverse roles in leaf senescence; for example, ethylene, ABA, SA, and JA accelerate senescence, but auxin and cytokinin delay it (Gepstein and Thimann, 1980; Noodén et al., 1990; He et al., 2002; van der Graaff et al., 2006; Kim et al., 2011; Khan et al., 2014). In addition to phytohormones, ROS-detoxification systems regulated by antioxidant enzymes also delay senescence by inhibiting ROS accumulation (Ye et al., 2000). Interestingly, ABA and SA induce ROS production in plant cells and also regulate stomatal closure (Jannat et al., 2011; Khokon et al., 2011). SA induces defense mechanisms (such as the hypersensitive response) against biotrophic pathogens and also triggers leaf senescence (Khan et al., 2014). Autophagy-defective mutants (*atg* mutants) exhibit hyper accumulation of SA, which accelerates programmed cell death and ROS accumulation in senescence (Yoshimoto et al., 2009). Exogenous auxin treatment also induces transient ROS production (Joo et al., 2001, 2005) and increases the transcript levels of *catalase* in maize (*Zea mays*) root cells (Guan and Scandalios, 2002). ROS not only function as second messengers to regulate signaling cascades, but they are also toxic molecules that induce apoptosis in plant cells in response to environmental stresses. ROS homeostasis, accompanied by the regulation of antioxidant enzyme activity, is also essential for plant development and the regulation of senescence (Ye et al., 2000; Lim et al., 2007; Procházková and Wilhelmová, 2007). Based on these findings, auxin-overproducing mutants may be exposed to high ROS levels induced by auxin and may therefore be sensitive to oxidative stress. However, *YUC6*-overexpressing plants, such as the dominant *yuc6-1D* mutant and *35S:YUC6* plants (both plants constructed under wild-type background), exhibit delayed leaf senescence and lower ROS levels in the presence and absence of oxidative stress (Kim et al., 2011, 2013). The delayed leaf senescence in *YUC6*-overexpressing plants results from the overproduction of auxin (Kim et al., 2011). More recently, we also found that YUC6 possesses unique TR and chaperone activity *in vitro* and that it exhibits FMO activity, which is necessary for auxin biosynthesis. To confirm these biochemical activities *in planta*, we transformed the *YUC6* or its mutant constructs in a wild-type Col-0 background harboring a *DR5:GUS* reporter gene, because a single loss-of-function mutant of biosynthetic genes did not display auxin deficient phenotypes due to its diversity of auxin biosynthesis pathway in *Arabidopsis* (Stepanova et al., 2011). Interestingly, we found that *YUC6*-overexpressing plants display enhanced drought tolerance caused by alterations in the induction of toxic ROS (Cha et al., 2015). These activities require the highly conserved Cys85 in YUC6. Thus, here we examined whether the Cys85-dependent TR activity of YUC6 also regulates leaf senescence.

Although the delayed senescence in *yuc6-1D* and *YUC6*-overexpressing plants results from their increased auxin levels, the regulation of ROS homeostasis by the TR activity of YUC6 may also cause this delayed senescence. Moreover, in the current study, we found that plants with a mutation of Cys85 in YUC6 did not exhibit delayed senescence under either natural or dark-induced senescence conditions (**Figures 1** and **2**). We previously showed that the YUC6-OX and YUC6-OX^C85S^ plants have similar auxin contents (Cha et al., 2015). However, the ROS contents of these plants differed during senescence; ROS levels were higher in YUC6-OX^C85S^ than in YUC6-OX plants (**Figure 3A**). These results indicate that the control of ROS levels is also essential for delaying YUC6-induced leaf senescence. However, it is still unclear why the YUC6-OX^C85S^ plants displayed accelerated leaf senescence, as their auxin levels were similar to those of YUC6-OX plants.

Based on recent findings, we investigated whether NTS and NGS are inactivated in YUC6-OX^C85S^ plants. NTS and NGS play important roles in redox balance, and a mutant with dysfunctional NTS and NGS pathways (*ntra ntrb cad2* triple mutant) displays a pin-like phenotype (Bashandy et al., 2010). These triple mutant plants are distinctly smaller than wild type at the rosette stage, with abnormal cotyledon and leaf shape and no floral structures. These phenotypes clearly indicate that these plants have reduced auxin transport activity, which is true for NTS and NGS mutant plants such as *cad2, ntra ntrb*, and *ntra ntrb cad2* mutants (Bashandy et al., 2010). In the current study, we also found that *NTRA, NTRB, NTRC*, and *GSH1*, which are involved in the redox system, were highly induced in YUC6-OX plants, and reduced in YUC6-OX^C85S^ plants, similar to wild type (**Figures 3B–E**). Therefore, the increased TR activity due to overexpression of *YUC6* activated genes involved in NTS and NGS. In addition, a chloroplastic NTRC mutant (*ntrc*) shows growth retardation with small and pale green leaves and reduced auxin levels under short-day conditions. This indicates that chloroplastic NTRC interacts with auxin to regulate development (Lepistö et al., 2009; Kirchsteiger et al., 2012). Thus, all three *Arabidopsis* NTRs regulating redox systems positively interplay with auxin in plant development. Interestingly, FAD and NADPH cofactor binding sites are well conserved in both YUC6 and TrxR proteins, and fully conserved Gly residues in both binding sites are necessary for the FMO and TR activity of YUC6 protein (Arnér and Holmgren, 2000; Jacquot et al., 2009; Cha et al., 2015). Furthermore, mutations of FAD or NADPH binding sites reduced auxin biosynthesis, delayed leaf senescence and drought tolerance in planta (Kim et al., 2011; Cha et al., 2015).

We previously determined that YUC6 regulates ROS homeostasis via its TR activity, which increases tolerance to oxidative and drought stresses (Cha et al., 2015). Bashandy et al. (2010) found that reducing thiol activity via BSO treatment (to inhibit GSH biosynthesis) disturbs auxin signaling including auxin levels and transport, indicating that the TR pathway regulates auxin homeostasis. Our findings also suggest that ROS accumulation induced by oxidative stress, like BSO treatment, reduces the expression of *PIN* genes (**Figure 4**). Increasing auxin levels via *YUC6*-overexpression activates auxin transporters, which produces auxin-induced phenotypes. By contrast, disrupting the TR activity of YUC6 via mutation of Cys85 disturbs ROS homeostasis and reduces the expression of auxin transporter genes (**Figure 5**). However, we did not observe phenotypic differences between YUC6-OX and YUC6-OX^C85S^ plants, which exhibit high-auxin phenotypes, although the levels of auxin transporter genes are limited in YUC6-OX^C85S^ compared to those in YUC6-OX (Cha et al., 2015). Perhaps the overexpression of *YUC6* in YUC6-OX^C85S^ plants continuously increases auxin biosynthesis, and spatial accumulation of auxin partly occurs by activation of auxin influx gene, *AUX1*, and main polar auxin eﬄux gene, *PIN1*. However, reduced TR activity of YUC6^C85S^ may perturb the control of ROS and reduce PIN levels to disturb auxin redistribution; this phenomenon requires further study.

## Conclusion

**FIGURE 6:**
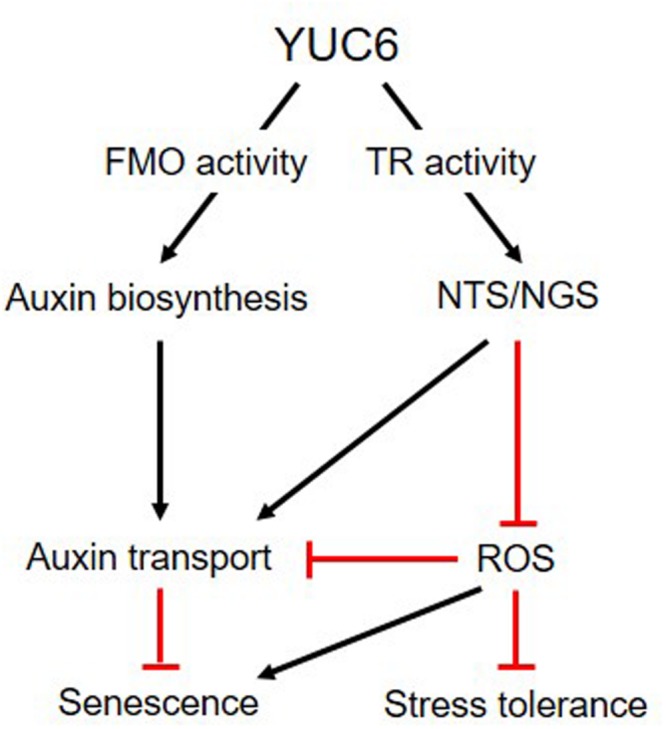
**Model linking the roles of YUC6 in auxin biosynthesis and ROS regulation.** YUC6 proteins exhibit *in vitro* FMO and TR activity involved in auxin biosynthesis and ROS homeostasis, respectively. *YUC6* overexpression increases auxin levels and triggers auxin transport throughout the plant, leading to auxin-induced developmental phenotypes. YUC6 also activates redox systems (such as NADPH-dependent thioredoxin and glutathione systems, termed NTS and NGS, respectively) via its TR activity, which is important for maintaining ROS homeostasis. These activated systems negatively regulate ROS accumulation caused by abiotic stress or senescence, but they positively regulate auxin transport. Thus, the TR activity of YUC6 is involved in both plant senescence and the acquisition of environmental stress tolerance via maintaining ROS homeostasis.

Our previous and current results show that YUC6 plays dual roles *in vitro*, displaying FMO and TR activities to activate auxin biosynthesis and NTS/NGS, respectively (**Figure 6**). Activated NTS/NGS positively regulates auxin transport and negatively regulates ROS production. Both of these activities, which are enhanced by *YUC6* overexpression, may influence leaf senescence. Taken together, these results suggest that the TR activity of YUC6 may trigger auxin transport and alter ROS homeostasis, leading to stress tolerance and delayed leaf senescence.

## Materials and Methods

### Plant Material and Growth Conditions

The open reading frames (ORF) of *Arabidopsis YUC6* (At5g25620) and its point mutation at Cys85 to Ser (*YUC6*^C85S^) were cloned into the pEarleyGate 101 vectors to overexpress *in planta*, introduced into *Agrobacterium tumefaciens* (GV3101), and then the construct re-introduced into a wild-type Col-0 background (harboring a *DR5:GUS* reporter gene) using floral-dip transformation method. Transgenic *YUC6*-overexpressing plants (YUC6-OX) and YUC6-OX^C85S^ *Arabidopsis thaliana* plants were selected using BASTA, and further confirmed their protein expression levels and auxin amounts displaying no differences among two overexpressing plants as described previously (Cha et al., 2015). Seeds were surface-sterilized with 30% bleach for 5 min, washed five times with sterile distilled water, and incubated for 2 days at 4°C. Plants were grown at 23°C on soil or on Murashige and Skoog (MS) medium containing 0.6% (w/v) agar and 20 g/L sucrose in Petri dishes under a 16 h/8 h light-dark cycle in a growth chamber.

### Senescence Assays

For the natural senescence assay, plants were grown on soil and monitored throughout their lives. For the dark-induced senescence assay, 3.5-week-old soil-grown plants were transferred to constant dark conditions at 23°C. Third and fourth rosette leaves were photographed at 7 days after dark treatment (7 DAT).

### Measuring Chlorophyll Contents

Chlorophyll contents were used as an indicator of senescence (Kim et al., 2011). Plants were subjected to dark-induced senescence as described above. Detached third and fourth rosette leaves were soaked in 80% (v/v) acetone, and total chlorophyll contents were measured by spectrophotometry as previously described (Ni et al., 2009).

### Detection of H_2_O_2_ by Histochemical Staining

H_2_O_2_ accumulation in plant cells was visualized using 3,3′-diaminobenzidine (DAB). Third and fourth rosette leaves of 40-day-old *Arabidopsis* plants grown in soil were detached and stained with DAB (1 mg mL^-1^, pH 3.8) for 4 h. The chlorophyll in leaves was removed by subsequent incubation in 80% (v/v) ethanol.

### Expression of PIN-GFP

Five-day-old *Arabidopsis* seedlings expressing *pPIN1:PIN1-GFP, pPIN2:PIN2-GFP, pPIN3:PIN3-GFP*, and *35S:GFP* were incubated in BSO (5 mM) or (10 μM) for 12 h. The plants were rinsed twice using distilled water, and GFP signals were analyzed by confocal microscopy (Olympus).

### Quantitative RT-PCR

For quantitative PCR, the third and fourth rosette leaves were harvested from 40-day-old (for **Figure 1**), 3.5-week-old (for **Figures 3B–E**), or 7 DAT plants after dark-induced senescence (for **Figure 2**). The transcript levels of auxin transporter genes *PIN1, PIN2, PIN3, PIN4*, and *AUX1* (as shown in **Figure 5**) were measured in 3.5-week-old seedlings. Samples were ground in liquid nitrogen, total RNA was extracted using TRIzol reagent (Qiagen), and cDNA was synthesized using oligo(dT) primer and reverse transcriptase (Solgent). Equal amounts of cDNA were used as templates for PCR amplification. Specific transcripts were amplified with gene-specific forward and reverse primers (Supplementary Table S1) using a step-cycle program and the Quantifast SYBR Green PCR kit (Qiagen). Quantitative PCR analyses were performed on three biological repeats. Amplification curves and gene expression levels were normalized to the expression of the housekeeping gene *TUBULIN*, which was used as an internal standard.

### Statistical Analysis

Statistical differences were calculated by two-tailed Student’s *t*-test, and one-way analysis of variance (ANOVA) followed by Duncan’s multiple range test applied for the calculation of confidence level at 95%.

## Author Contributions

J-YC, D-JY, and W-YK initiated the project. J-YC, MK, IJ, SK performed the experiments. J-YC, D-JY, and W-YK analyzed the data. J-YC, HP, MK, W-YK wrote the paper with input from other authors. All authors discussed the results and approved the manuscript.

## Conflict of Interest Statement

The authors declare that the research was conducted in the absence of any commercial or financial relationships that could be construed as a potential conflict of interest.
