# Supplementary material for: The Thiol Reductase Activity of YUCCA6 Mediates Delayed Leaf Senescence by Regulating Genes Involved in Auxin Redistribution
Source: Front Plant Sci. 2016 May 9;7:626. doi: 10.3389/fpls.2016.00626 (PMC4860463; doi:10.3389/fpls.2016.00626)
Supplement: Supplementary file 1 [file Table_1.PDF]

Supplementary Table S1. Primer sequences used in this study.

Name	Primer (5′->3′)
SAG12<br>(AT5G45890)	F: GTTAATGATGAGCAAGCACTG<br>R: GGAAATCAAAACCACCTCC
NTRA<br>(AT2G17420)	qRT F: TTGGAGGATTGAAGGTGAAGAA<br>qRT R: GGCTTGGTCACAACATAACCAT
NTRB<br>(AT4G35460)	RT F: CGAAAGCTTTGCACGGCTTGGTGGTG<br>RT R: GATCAATCAACAATAACTCAATGACCT
NTRC<br>(AT2G41680)	qRT F: GAGGAAGCCTTGTATCTCACGAAA<br>qRT R: CTGATCTCTGCGAACAAGCAAA
GSH1<br>(AT4G23100)	F: GATTTGACATAGTGTAATGTG<br>R: AGTCAGAATATTAATCTAACC
AUX1<br>(AT2G38120)	RT F: GAAGGGAGTAAAAGTCACG<br>RT R: AGGAGAGAAGACGATACAT
PIN1<br>(AT1G73590)	RT F: TGGGATGATGAATTGTGAAAAG<br>RT R: CCAAAAGAGGAAACACGA
PIN2<br>(AT5G57090)	RT F: GGCGATACGACCCAAA<br>RT R: TGTACTTGCACATCCCAAA
PIN3<br>(AT1G70940)	RT F: GAATGCTGATGCCAACA<br>RT R: AGAGTTACCCGAACCTAAT
PIN4<br>(AT2G01420)	RT F: CCACTTCATCTCCACCAACGATCC<br>RT R: CCGGACCAAAATTCGAAAGCCTCC
